# American Society of Dental Surgeons

**Published:** 1851-10

**Authors:** 


### EDITORIAL DEPARTMENT.

*American Society of Dental Surgeons.*—As we were prevented from attending the last meeting of the American Society of Dental Surgeons, held the 5th, 6th and 7th of August, in Philadelphia, we are indebted for the information we have, concerning the transactions of the occasion, to the courtesy of the secretary, Prof. Cone, who has kindly permitted us to examine the minutes of the proceedings, and to a brief abstract of the doings furnished by one of the members. The reports of committees and addresses read at the meeting, have also been placed in our hands by the chairman of the publishing committee, for publication; most of which will be found in another part of the present number of the Journal. The Society met at Sanson-street Hall at 9 o'clock, Tuesday, August 5th, and after having been called to order by the president, Dr. E. Parmly, the dentists of Philadelphia were, in accordance with a resolution offered by Dr. E. Townsend, invited to attend the discussions of the society during the meeting. The order of the business for the session was then announced by Dr. J. H. Foster of New York, chairman of the executive council.

During the morning session of the first day, the publishing committee reported that the Aphorisms ordered at the preceding meeting, had not been placed in their hands. The treasurer's report was read and referred to a committee to be audited. The members appointed at the meeting held in 1850, to prepare Aphorisms on Specified Subjects, on being called on, informed the society, that they had performed the duty assigned them.

It will be recollected that at the preceding meeting, a resolution was offered, calling for the appointment of six members, each to prepare a series of aphorisms on a specified subject, and that when prepared, to be placed in the hands of the publishing committee for approval and immediate publication, with six other series which had been previously prepared. But as the last six, had not been placed in the hands of the committee before the meeting, Dr. Townsend moved, that they be submitted to the society for its approbation previously to their publication.

Dr. W. O. Laird's name, agreeably to his request, communicated by letter to the secretary, was stricken from the list of members. A committee was then appointed, on motion of Dr. Dunning, to confer with the treasurer, and report the names of such members, as had forfeited their membership by the non-payment of their yearly dues. Another committee was appointed, to take into consideration the propriety of altering the constitution of the society, with instructions to report such alterations and amendments as might be deemed necessary. This committee, composed of two members, Drs. Townsend and Arthur, has, by subsequent action of the society, until the next meeting, to make its report. We sincerly trust, the subject entrusted to it, will receive that calm and deliberate attention its importance demands. There are many parts of the constitution which require alteration, and new provisions should be introduced.

Wednesday the 6th, at 4½ P. M., having been fixed upon as the time for the delivery of the opening address by Dr. Foster, the secretary, on motion of Dr. Townsend, was instructed to invite the public to be present on the occasion, the invitation to be published in two of the daily papers.

The Aphorisms of Dr. Dunning, on the treatment of exposed dental nerves, were next read. As we have not seen the series, we are ignorant of the views which they embody, but learn from the proceedings of a subsequent session, that the two first were amended so as to read, "a tooth, whose nerve is exposed, may be permanently saved, by entirely removing the pulp, and filling the place occupied by it with gold."

We presume we understand the idea intended to be conveyed by this Aphorism, but we fear the impression it will make upon the minds of most persons, will be different from that intended by the society. We suppose the meaning of the Aphorism to be this, that a tooth, the nerve of which is exposed, may, in the majority of cases, or often, be saved for many years, and sometimes through life. As it now reads, it may be taken without any qualification, and if an individual into whose hands it may fall, should have a tooth treated in this manner, and if, perchance, it should ever give rise to alveolar abscess, or other diseased action in the surrounding parts, requiring for its cure, the removal of the organ, the judgment of the society would ever after be held in light estimation by that individual. If the mover of the amendment, or the society, had reflected a moment upon the subject, we think some qualifying word would have been introduced, whereby the meaning of the Aphorism could not have been misconstrued.

During the afternoon session of the first day, Dr. A. C. Hawes, read a series of Aphorisms on the preservation of the teeth, and Dr. A. Hill, a report of the result of his experiments for obtaining metallic bases, for artificial teeth, by the electro-galvanic process, which was followed by some remarks from Prof. J. Allen, of Cincinnati, upon the same subject. Prof. A. then went on to speak of some experiments which he had made for securing single teeth to plates, by means of a fusible mineral attachment, which had thus far proved highly satisfactory to himself. He also exhibited several pieces illustrating his discovery. This method of mounting teeth, has been practiced in France for about thirty years, and there is now in the museum of the Baltimore College of Dental Surgery, a double set of teeth, presented to the Institution, by Dr. Townsend of Philadelphia, mounted in this manner. They were made some nineteen or twenty years ago.

The committee appointed to confer with the treasurer, and report the names of such members as had forfeited their membership by the non-payment of their dues, now made their report, when a resolution was offered, ordering the names of such members to be stricken from the roll.

During the forenoon session of the second day, Dr. A. Hill, read an essay on artistic dentistry, and Dr. A. C. Hawes, a paper on professional empiricism. The remainder of the session, was taken up in the transaction of private business, and in the discussion of the subject of the Aphorisms, on the treatment of exposed dental nerves, in which Drs. Arthur, Westcott, Dunning, Allen, White, Bridges, Foster, J. Parmly, Cone, Hill and Robertson, took part.

In the afternoon, Dr. J. H. Foster, delivered the annual oration, which, we understand, was pronounced, by all, to be an able production. This we have not seen, but hope to have an opportunity of placing it before our readers.

This day, the society had an evening session, during which, Dr. Arthur read a report on American dental literature, after which Dr. Townsend read a paper on the preservation of the temporary teeth, and the treatment of irregularity of the permanent ones, which gave rise to some discussion.

A portion of the session of the morning of the third day, was taken up in the transaction of private business; after which, Dr. E. Townsend, proposed the following amendment to the constitution, reading from the one first adopted, Art. 5, "Of the requisition of membership. Sec. 1. Each and every acting member of this society, shall either have become such, by virtue of his attendance in person, by proxy, or by letter, at the time of its formation, or as shall be afterwards elected, as shall be prescribed by the constitution, and subscribed to the same," *Provided,* that all delegates appointed to represent recognized dental colleges, and associations of dental practitioners, organized for scientific purposes, shall be admitted to all the rights and privileges of acting members of this society, as fully as if they had been elected to such membership by ballot, under the provision of Art. 6th, of this constitution.

The above amendment, Dr. T. supported by the following argument, read to the society, and a copy of which has been kindly furnished us for publication.

"Mr. President—I have offered this amendment to our constitution, for the purpose of supplying a provision which is so completely within its scope and purpose, that its absence seems a mere oversight in the original draft of the instrument. The election, by ballot, which is the prescribed mode of admission to membership in all cases, as the constitution now stands, is, of course, but a method of ascertaining the worthiness of the applicant, who, in all cases, except those contemplated by the amendment now submitted, comes to us without other warranty than his personal or individual character, upon which it is right and necessary that this society should make up a deliberate judgment before admitting him, and thereby endorsing him. But delegates from recognized dental colleges and associations, in other words, bodies of our professional brethren which we acknowledge and respect, come to us with credentials which we are bound to honor as much as we ask other bodies to respect and honor our own. And it is a violation of right and propriety to make such delegates wait at our doors for admission, after they have sent in their cards and letters of introduction, while we go through the form of rejudging the judgment of the men who sent them. We certainly would not willingly have our trusted and honorable representatives so received, by any society of equals in the world, and I know we would not send representatives to any society which held itself our superior. When a respectable association of scientific men have selected delegates, to any other with whom they are willing to enter into professional reciprocities, to examine in any way, or to subject to any test, the worthiness of such delegates, is to disregard and discredit those who gave them their appointment.

"I need say no more on this point, except to add, that the amendment in effect, does nothing but allow the endorsement of respectable societies and colleges to pass unquestioned with us; and members so admitted, will come in, subject to all the conditions and entitled to all the privileges, and none other, that attach to those admitted by our present mode of initiation. They will stand to us precisely as if we had unanimously elected them, and it will remain with them as with others, to sign the constitution, and comply with its provisions, in order to entitle them to equal participation with us in all that pertains to full membership in our society. Mr. President—Mere verbal or ceremonial amendments to constitutions are not worthy of the time they occupy, and I would not propose one, unless I thought the *words* which I suggest meant *things*, and the *forms* stood for important substances. We *style* ourselves 'The *American* Society of Dental Surgeons,' and our objects, as they are stated in the preamble to the constitution, are, among others, 'to promote union and harmony among all respectable and well informed dental surgeons; and to advance the science by free communication and interchange of sentiment, either written or verbal, between members of the society, both in this and other countries.' Thus the name and the attitude which we take, assume to fraternize the men, and centralize the movement by which our profession is to be carried forward to its destined position in the world of science; and it is incumbent upon us to adjust and address ourselves to our function by every proper means that promises to promote our aim.

"The adoption and publication of this amendment, in connection with our other action on kindred points, I conceive, will indicate the liberal and cordial spirit which animates us, and will be received as an earnest overture to the professional brotherhood everywhere, for that correspondence and co-operation which must answer to the achievement of our great design. Eleven years ago our society rose out of the crowd of professors, and pretenders to dental skill, for a purpose which happily stands well accomplished now, whatever agencies may take the credit of it. I mean, that it drew a line of distinction between the rabble of quacks and the men of honest pretensions, expecting that the profession would soon be found *within* its membership, and the charlatans *without*. Or, at least, that by its effort to help all other agencies, a standard would soon be established for public judgment which would render honor to whom honor was due, and rid the profession of all the responsibility and discredit of empiricism; now this work is well enough accomplished to answer all the ends for which it was undertaken, and the attitude of offensive and defensive war should be changed in all points, so that no prejudice or suspicion shall be allowed to remain, that we are taking more care of our character and caste, than of the *other* great interests of the profession.

"Mr. President—A society is like other creations, made of live materials, where it ceases to grow, it begins to die; not consciously, perhaps, but none the less certainly. The laws of the human body well represent the laws of human societies—whenever the *old* ceases to be replaced by the *new,* when the work of assimilation and the incorporation of fresh elements stops the body, or the society is on the turn; and decline once begun, means death for its conclusion. Churches need revivals, and political governments occasionally refresh themselves even with revolution, scientific societies also demand renewal in spirit and in *men,* but they can secure it by providing for their regular growth and constant accommodation to the changed conditions around them by corresponding changes within them.

"I know I do not mistake the feelings of this association, and I am sure I understand its objects—they are both liberal and enlightened—and they aim earnestly at the greatest advancement for our profession which they can contribute to secure.

"Let us manifest, by every means within the compass of our power, that we design and desire the union, harmony and co-operative activity of every worthy worker in the cause of progress, that we have no jealousies of rank and no privileges of age to defend, that we do not wish to establish a close corporation of exclusives, but a liberal republic of dental literature and science—we might invite delegates in words of respect and welcome, or wait until they offered themselves and then admit them to the courtesies of our annual meetings, but such men as there is any interest or honor in inviting would not come to us as guests to receive our hospitalities, they would come and we would receive them as equal members, on the level platform of a broad brotherhood that exchanges honors and benefits evenly. There are dental colleges and dental societies now in several regions of our own country, and there are some associations abroad, whom we would warmly welcome into friendly correspondence and fellowship of effort, and we will have done our duty in the matter, by opening our doors widely for their admission. The 15th article of the constitution requires a majority of three-fourths of all the active members or fellows present to carry an amendment. I am flattering myself, sir, with the hope of an unanimous vote for this one, which I have the honor to propose."

The amendment and argument were, by a vote of the society, laid on the table.

With regard to the propriety and expediency of engrafting Dr. T's provision into the constitution of the society, we have not now time to express an opinion. We may hereafter recur to the subject.

The aphorisms of Dr. E. Parmly, were taken up, and adopted, article by article. It would seem, from a resolution of Dr. J. D. White, that the society intended to discuss the cause and treatment of irregularity of the teeth, but deferred it until the next meeting, and on motion of Dr. M. K. Bridges, one member was appointed from each of the principal cities, to investigate the subject and report at that time. This committee is composed of Drs. M. K. Bridges, E. G. Tucker, J. D. White, J. Allen and C. O. Cone.

Drs. J. D. White and E. Townsend, were appointed a committee to make microscopical observations on the characteristics of salivary calculus and the fluids of the mouth, in connection with the human teeth, and to report at the next meeting of the society.

Various subjects connected with the practice of dental surgery were discussed by several members of the society, after which, the following gentlemen were elected officers for the ensuing year:

Dr. E. Parmly, of New York, President; Dr. E. Townsend, 1st Vice President; Dr. J. H. Foster, 2d Vice President; Dr. J. Tucker, 3d Vice President; Dr. C. O. Cone, Corresponding and Recording Secretary; Dr. E. J. Dunning, Treasurer; Dr. D. R. Parmly, Librarian; Drs. C. O. Cone, E. J. Dunning and D. R. Parmly, Publishing Committee; Drs. J. H. Foster, E. J. Dunning, E. Parmly, A. C. Hawes, C. A. Harris and H. N. Fenn, Executive Counsel and Examining Committee.

Dr. Arthur was appointed to deliver the opening address at the next annual meeting, and Drs. J. D. White, E. Townsend, E. G. Tucker, J. Allen and A. Robertson, to read essays on subjects connected with the theory or practice of dental surgery; Dr. C. A. Harris was appointed a committee on foreign dental literature, to report at the next annual meeting.

We learn that Dr. Williams, of Philadelphia, was elected a member of the society.

The society adjourned to meet the first Tuesday of August, at the Ocean House, Newport, R. I.

In addition to the foregoing summary of proceedings of the society, we take pleasure in publishing the following interesting notice, kindly furnished by one of the members who attended the meeting.

*Twelfth Annual Meeting of the American Society of Dental Surgeons.*— The annual meeting of this association was held in Philadelphia on the first Tuesday in August. A brief abstract of the proceedings will be found in another part of the present number of the "Journal."

This has, altogether, been the most agreeable and profitable meeting of the association it has yet been our privilege to attend. More profitable, probably, in its suggestions, than in the actual amount of new facts presented, although amongst these were some of considerable value.

A liberal spirit toward the profession at large, without regard to position, was manifested in the beginning, and circulars of invitation to attend its meetings were sent to most of the resident dentists of Philadelphia. A number responded to the invitation and attended the meetings of the association to the close.

A number of interesting papers were read by the several members who were appointed at a former meeting to prepare them. These will, in due time, be published in the Journal. Without wishing to be invidious, for all had real merit, we desire to notice the opening address of Dr. Foster.

This was an able production, and marked with manliness and a spirit of true liberality—it did not run off into that other extreme which, though frequently well meant, tends rather to countenance the dishonest in this bad practice, than to visit upon them that just condemnation which they deserve, and which it is due to the public that they should receive.

A very interesting discussion on the operation of removing the dental pulp sprung up on a series of aphorisms offered by Dr. Dunning on the subject. The results of, and the objections to the use of arsenic as an adjunct for the purpose, were freely canvassed. The discussion was discontinued for want of time, and not by any means because the subject was exhausted.

The subject of irregularities of the teeth also came up, and a good deal of new light was thrown upon this very important branch of our art, by the gentlemen who took part in the discussion. It, also, was cut off for want of time, and it was agreed that at the next annual meeting every member who could do so, should keep a record of instructive cases, which might come into his hands in the interim, and bring with him models to enable him to explain his mode of treatment. We look forward to the result of this effort to obtain some general views with regard to this matter with much interest. There is no subject within the range of our profession so little understood generally as this, and much good must result from this combined effort to throw light upon it.

Dr. Hill, who was appointed for the purpose of making experiments to ascertain whether it was feasible to produce plates by the electro-metallic process, in a manner which would be practically serviceable, exhibited several plates made in this way. They were deposited on the plaster cast. Dr. Allen, of Cincinnati, also exhibited several very beautiful specimens of plates formed in this way, and stated that several plates made in the same manner were now worn by his patients. Neither gentlemen, however, seemed to think, judging from the experiments he had made, that the process was of much practical value for this purpose. The principal objection seemed to be, that too much time was required to form a perfect plate, which Dr. Allen stated to be some seven or eight days.

Dr. Allen exhibited some specimens of a new method of mounting mineral teeth. On examining the sets of teeth, which he exhibited, they presented somewhat the appearance of block gum teeth, without any visible means of fastening. They were, however, firmly fixed to the plate. Dr. A. stated that the improvement consisted in a material he had discovered, which fused at a white heat without shrinking, attaching itself at the same time to the teeth and plate. Ordinary single plate teeth are used and arranged in the usual way; when they are properly adjusted, some of the material he uses, is moulded around them neatly, so as to form a gum, and to fill all the spaces between the teeth and plate. The whole is then put into the furnace and subjected to a high heat—the material between the teeth and plate fuses and attaches itself so firmly, that the teeth will break before the connecting material will give way. As the plate is subjected to this high heat, it is alloyed with platina, to prevent it from melting. On being asked what was the material used, Dr. A. declined stating, alleging that the proper means of successfully using it, could only be acquired by personal instruction, which, for a just compensation for his time and labor, he was disposed to give. Dr. A. said he was opposed to taking out a patent for improvements in the profession, and although a caveat had been filed in the patent office for this particular improvement, it might be there forever. But as he had spent a great deal of time and labor and thousands of dollars to accomplish this object, he thought it but right that he should have some return for it. This he only desired, as we have stated in the way of compensation for instruction, which he was ready to give.

Since the meeting of the association, we have heard, through a friend, that Dr. Hunter of Cincinnati, declares that he has a better material than that which was exhibited to the association by Dr. Allen, and that he will in a short time publish the method of preparing it.

In a short familiar discussion, which sprung up on the best manner of preparing gold for filling teeth, Dr. Arthur stated that for some time past he had for all kinds of cases used gold known as No. 30, with great advantage. It is used in this form by cutting it from the sheet in a single strip, nearly the size of the cavity to be filled, and is carried into the cavity with instruments of the usual form. This is stated to be an important feature in its use, for if folded or rolled in the usual way, it will be perfectly unmanageable.

Amongst others, a very important committee was appointed for the purpose of making microscopical investigations of the teeth in a healthy and diseased condition, and also of the fluids of the mouth and the tartar. It is certainly quite time that we began to do something in this way for ourselves; we have heretofore been entirely dependent upon European laborers for all we know of the internal structure of the teeth, and have not even done so much as will enable us to judge of the real value of their researches. This committee consists of Drs. J. D. White and E. Townsend, of Philadelphia, gentlemen fully able to take up this important subject. They will probably have use of the best apparatus for the purpose in this country; and the profession will look forward with interest to the result of their labors.

It would afford us much pleasure to present an abstract of the discussion which took place at this meeting of the association, if time and space permitted. The greater part of them, however, were taken down by a reporter and will be published.

The meeting adjourned on Friday afternoon, to meet at Newport, at the usual time, next August.

We cannot but congratulate the members of this association upon the delightful spirit which prevailed from beginning to end, at this meeting. All personal bickerings, which so often disfigure and destroy the usefulness of meetings of this character, in other professions, as well as our own, were avoided, and every man seemed to be looking rather to the general good of the profession, than considering his own personal advancement. The only regret seemed to be, that the time of separation came so soon.

